# Establishment of a novel amyotrophic lateral sclerosis patient (*TARDBP* ^N345K/+^)-derived brain microvascular endothelial cell model reveals defective Wnt/β-catenin signaling: investigating diffusion barrier dysfunction and immune cell interaction

**DOI:** 10.3389/fcell.2024.1357204

**Published:** 2024-08-15

**Authors:** Kinya Matsuo, Jun Nagamatsu, Kazuhiro Nagata, Ryusei Umeda, Takaya Shiota, Satoru Morimoto, Naoki Suzuki, Masashi Aoki, Hideyuki Okano, Masayuki Nakamori, Hideaki Nishihara

**Affiliations:** ^1^ Department of Neurology and Clinical Neuroscience, Yamaguchi University Graduate School of Medicine, Yamaguchi, Japan; ^2^ Yamaguchi University Graduate School of Medicine, Yamaguchi, Japan; ^3^ Keio University, Regenerative Medicine Research Center, Kawasaki, Kanagawa, Japan; ^4^ Department of Neurology, Tohoku University Graduate School of Medicine, Sendai, Japan

**Keywords:** blood-brain barrier, amyotrophic lateral sclerosis, TDP-43, human induced pluripotent stem cells, Wnt/β-catenin signaling

## Abstract

Amyotrophic lateral sclerosis (ALS) is a major neurodegenerative disease for which there is currently no curative treatment. The blood-brain barrier (BBB), multiple physiological functions formed by mainly specialized brain microvascular endothelial cells (BMECs), serves as a gatekeeper to protect the central nervous system (CNS) from harmful molecules in the blood and aberrant immune cell infiltration. The accumulation of evidence indicating that alterations in the peripheral milieu can contribute to neurodegeneration within the CNS suggests that the BBB may be a previously overlooked factor in the pathogenesis of ALS. Animal models suggest BBB breakdown may precede neurodegeneration and link BBB alteration to the disease progression or even onset. However, the lack of a useful patient-derived model hampers understanding the pathomechanisms of BBB dysfunction and the development of BBB-targeted therapies. In this study, we differentiated BMEC-like cells from human induced pluripotent stem cells (hiPSCs) derived from ALS patients to investigate BMEC functions in ALS patients. *TARDBP* ^N345K/+^ carrying patient-derived BMEC-like cells exhibited increased permeability to small molecules due to loss of tight junction in the absence of neurodegeneration or neuroinflammation, highlighting that BMEC abnormalities in ALS are not merely secondary consequences of disease progression. Furthermore, they exhibited increased expression of cell surface adhesion molecules like ICAM-1 and VCAM-1, leading to enhanced immune cell adhesion. BMEC-like cells derived from hiPSCs with other types of *TARDBP* gene mutations (*TARDBP* ^K263E/K263E^ and *TARDBP* ^G295S/G295S^) introduced by genome editing technology did not show such BMEC dysfunction compared to the isogenic control. Interestingly, transactive response DNA-binding protein 43 (TDP-43) was mislocalized to cytoplasm in *TARDBP* ^N345K/+^ carrying model. Wnt/β-catenin signaling was downregulated in the ALS patient (*TARDBP* ^N345K/+^)-derived BMEC-like cells and its activation rescued the leaky barrier phenotype and settled down VCAM-1 expressions. These results indicate that *TARDBP* ^N345K/+^ carrying model recapitulated BMEC abnormalities reported in brain samples of ALS patients. This novel patient-derived BMEC-like cell is useful for the further analysis of the involvement of vascular barrier dysfunctions in the pathogenesis of ALS and for promoting therapeutic drug discovery targeting BMEC.

## 1 Introduction

Amyotrophic lateral sclerosis (ALS) is a fatal neurodegenerative disease in adults, affecting primary and secondary motor neurons, causing progressive limb weakness, dysphagia, and respiratory paralysis (Feldman et al., 2022). While most ALS cases are sporadic, 5%–10% are familial, with nearly 30 causative or modifying genes identified, including *SOD1*, *C9ORF72*, *FUS*, and TAR DNA-binding protein gene (*TARDBP)* (Gregory et al., 2020). *TARDBP* encodes the protein transactive response DNA-binding protein 43 (TDP-43), which binds to both DNA and RNA and regulates their expression and splicing. Aggregation of TDP-43 in ubiquitin-positive cytoplasmic neuronal inclusions, even in sporadic ALS cases without *TARDBP* variants, is now recognized as a pathological hallmark of the disease.

Although motor neuron degeneration occurs in the central nervous system (CNS), accumulating evidence suggests an association between neurodegeneration and activation of the peripheral immune system and humoral factors in the blood. For example, a cohort study of ALS patients reported that elevated C-reactive protein (CRP) in peripheral blood correlates with predominantly impaired motor function and reduced life expectancy (Lunetta et al., 2017). The population of specific peripheral immune cell subsets in peripheral blood fractions of ALS patients linked to their functional state and prognosis (Murdock et al., 2017; Cui et al., 2022; Grassano et al., 2023). In addition, blood-derived molecules, such as fibrins, and thrombin could be critical elements leading to neuroinflammation and neurodegeneration (Ryu et al., 2018; Shlobin et al., 2021; Finney et al., 2023). Indeed, the presence of serum components and peripheral immune cells such as T-cells, macrophages and natural killer cells within the spinal cord, motor cortex, and brainstem of autopsy samples from ALS patients have been repeatedly reported (Troost et al., 1989; Troost et al., 1990; Kawamata et al., 1992; Engelhardt et al., 1993; Graves et al., 2004; Henkel et al., 2004; Garbuzova-Davis et al., 2012; Winkler et al., 2013; Jara et al., 2019; Garofalo et al., 2020; Garofalo et al., 2022). These data strongly suggest that humoral factors in serum and/or immune cells appear to be important drivers of neurodegeneration. In physiological conditions, however, the CNS is a sanctuary separated from peripheral tissues by the blood-brain barrier (BBB), which is a specific series of functions of the brain vasculature and plays an important role in maintaining CNS homeostasis. Not a single cell type, but several cellular and non-cellular components, such as brain microvascular endothelial cells (BMECs), pericytes embedded in vascular and parenchymal basement membranes, astrocyte endfeet, microglia, and neurons, are involved in vascular barrier properties and are referred to as the neurovascular unit (NVU). Compare to endothelial cells in peripheral organ, BMECs are unique in having continuous tight junctions, no fenestrae, and specific polarized transporters and efflux pumps. BMECs also regulate immune cell migration into the CNS by limiting the expressions of endothelial adhesion molecules on their surface (Engelhardt and Ransohoff, 2012). Under certain pathological circumstances, the expression of these molecules, such as intercellular adhesion molecule-1 (ICAM-1) and vascular cell adhesion molecule 1 (VCAM-1), is upregulated, leading to increased and uncontrolled immune cell entry into the CNS. Given the fact that BBB maintains homeostasis of the CNS, studying BMEC dysfunction in ALS is an attractive target to identify novel disease pathomechanisms and develop potential therapeutic targets.

Recently, *TARDBP* mutations have been focused on to elucidate the molecular pathway underlying vascular barrier dysfunctions in ALS. Animal studies have suggested that *TARDBP* mutation and/or alteration of TDP-43 expression lead to diffusion barrier dysfunction and aberrant immune cell infiltrations (Sasaki et al., 2015; Zamudio et al., 2020; Cheemala et al., 2023). Notably, RNA transcripts and nuclear proteins analysis showed that the brain capillary endothelial cells from patients with ALS or frontotemporal lobar degeneration (FTLD) expressed reduced levels of TDP-43 and nuclear β-catenin along with diminished expression of canonical Wnt response genes, which is important for BBB development and maintenance (Omar et al., 2023). In addition, knockdown of TDP-43 or overexpression of aberrantly truncated TDP-43 in mouse brain endothelial cells results in reduced tight junction protein expression (Xu et al., 2022). These reports indicate that TDP-43 plays a regulatory role in BMEC function and physiological dysfunction of TDP-43 is associated with BMEC alterations in ALS patients. Despite the growing interest in the relationship between TDP-43 and vascular barrier function, the structural and functional differences in TDP-43 between species have prevented the demonstration of direct involvement of TDP-43 in vascular barrier abnormalities in humans (Wang et al., 2004; Ling et al., 2015). This highlights the need for detailed analysis using ALS patient-derived models.

We have recently established the method called extended endothelial cell culture method (EECM) for differentiating BMEC-like cells from human induced pluripotent stem cells (hiPSCs) and investigated the role of the BMEC in pathological conditions (Nishihara et al., 2020; Nishihara et al., 2021; Nishihara et al., 2022; Matsuo et al., 2023). Previous studies have reported that hiPSC-derived neurons and astrocytes reflect parts of ALS pathophysiology and therapeutic effect (Okano et al., 2020; Okano and Morimoto, 2022; Morimoto et al., 2023; Okano et al., 2023; Leventoux et al., 2024; Thiry et al., 2024), but study about endothelial cell functions in ALS is limited (Katt et al., 2019). The strength of our BMEC-like cell is that it exhibits pure endothelial characteristics in morphology and transcriptomes, and has both proper diffusion barrier function and adhesion molecule expression, making it unique and suitable for studying the morphological changes and link between the peripheral immune system and the CNS across the BBB (Nishihara et al., 2020; Gastfriend et al., 2021a).

In this study, we presented the successful establishment of BMEC-like cells from iPSCs derived from an ALS patient carrying the *TARDBP* ^N345K/+^ as an *in vitro* ALS-BMEC model. Furthermore, we generated a BMEC-like cells from two genome-editing hiPSC clones targeting *TARDBP* ^G295S/G295S^ and *TARDBP* ^K263E/K263E^ from the healthy donor-derived hiPSC and investigated whether the specific *TARDBP* mutations contribute to BMEC dysfunction. ALS patient (*TARDBP* ^N345K/+^)-derived EECM-BMEC-like cells showed impaired diffusion barrier functions and enhanced adhesion molecule expression, resulting in increased immune cell adhesion. In addition, TDP-43 cytoplasmic deposition and downregulated Wnt/βcatenin signaling pathway was observed in *TARDBP* ^N345K/+^ carrying BMEC-like cells. Furthermore, their leaky barrier phenotype and elevated VCAM-1 expressions were ameliorated by the activation of Wnt/β-catenin signaling. These results demonstrate that novel ALS patient-derive BMEC-like cells are useful for studying the mechanisms of how TDP-43 affects BMEC function and the identification of therapeutic strategies targeting BBB.

## 2 Material and method

### 2.1 Donors and human induced pluripotent stem cells (hiPSCs)

**FIGURE 1:**
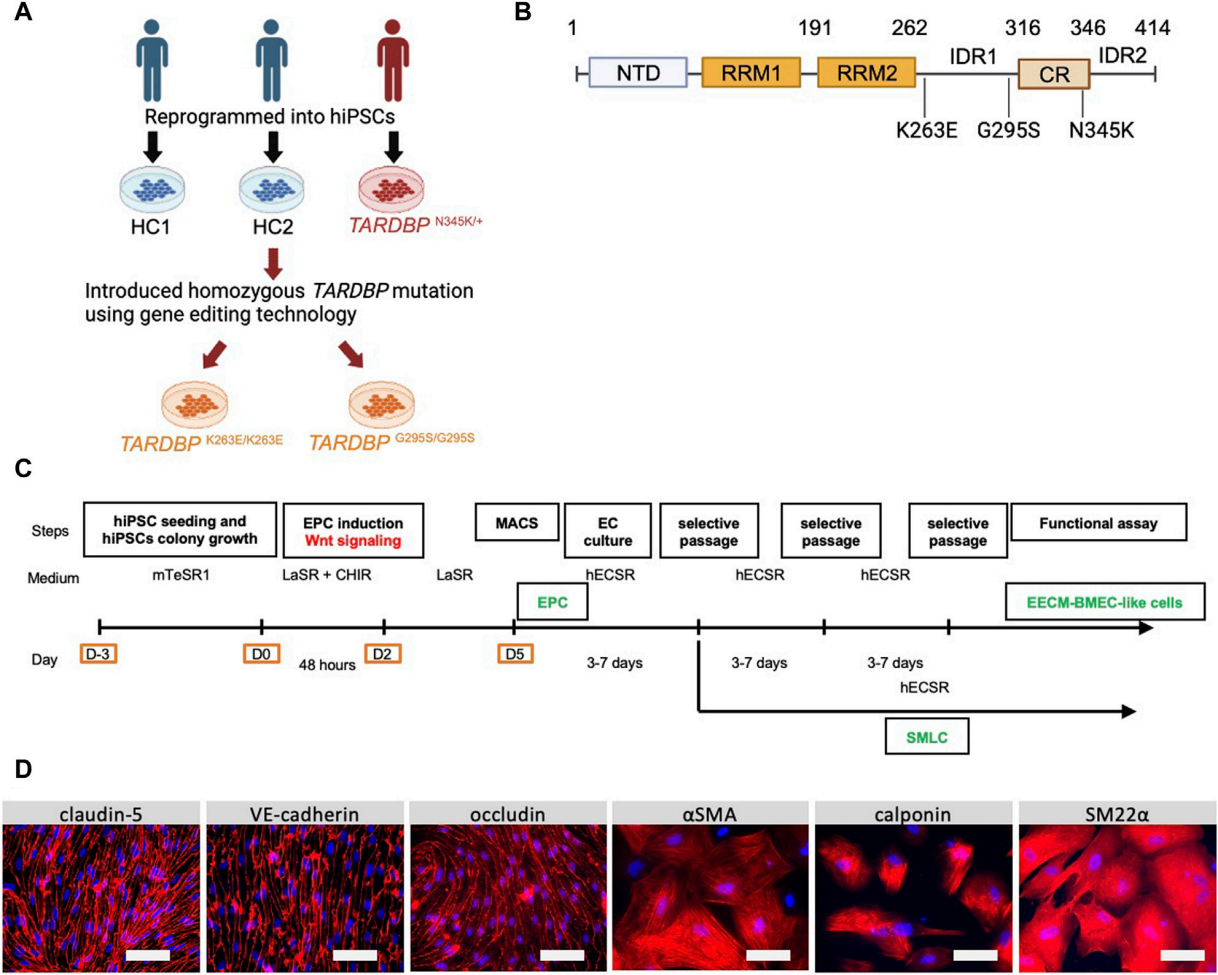
Establishment and characterization of EECM-BMEC-like cells and SMLCs. **(A)** An overview of the donors of hiPSCs and the origin of *TARDBP* mutated gene editing hiPSC clones were shown. hiPSCs were generated from two distinct healthy donors (HC1 and HC2) and one ALS patient carrying heterozygous *TARDBP* mutation, *TARDBP* ^N345K/+^. HC2 is the isogenic control of *TARDBP* ^K263E/K263E^ and *TARDBP* ^G295S/G295S^. hiPSC: human induced pluripotent stem cells, HC: healthy control, ALS: amyotrophic lateral sclerosis. Figure created with BioRender.com. **(B)** The Locus of each *TARDBP* mutation was shown. NTD: N-terminal domain, RRM: RNA recognition motif, IDR: intrinsically disordered regions; CR: conserved region. Figure created with BioRender.com. **(C)** The overview of EECM protocol. EPC: endothelial progenitor cell, MACS: magnetic activated cell sorting, EC: endothelial cell, mTeSR1/LaSR/CHIR/hECSR: specific name of the medium and compound. Recipes were shown in Supplementary Table S2. EECM: extended endothelial cell culture method, BMEC: brain microvascular endothelial cell, SMLC: smooth muscle-like cell. **(D)** Representative immunostaining images of EECM-BMEC-like cells and SMLCs. Cells from HCs were grown in an 8-well chamber plate and EECM-BMEC-like cells were stained for junctional protein VE-cadherin, claudin-5, or occludin (red). SMLCs were stained for smooth muscle cell markers αSMA, calponin, or SM22α (red). Nuclei were stained using DAPI (blue). Scale bar = 50 μm.

The donors of hiPSCs and the origin of the *TARDBP*-mutated gene-edited hiPSCs were summarized in Figure 1A. A patient-derived hiPSC line was reprogrammed from T lymphocytes derived from a patient with familial ALS (age/sex: 63/M, *TARDBP* p.N345K) (Leventoux et al., 2020). The isogenic hiPSC lines that induced two distinct pathogenic mutations of the TDP-43 protein, *TARDBP* ^K263E/K263E^ and *TARDBP* ^G295S/G295S^, were generated as described (Imaizumi et al., 2022). Both of the mutations have been reported to be associated with ALS and FTLD (Floris et al., 2015) and the locus of each mutation is shown in Figure 1B. Two healthy controls (HC) derived hiPSC lines HPS1006 and HPS4290 (Takahashi et al., 2007) (age/sex: 36/M, 36/F, respectively) were provided by the RIKEN BRC through the National Bio-Resource Project of the MEXT/AMED, Japan. *TARDBP* ^K263E/K263E^ and *TARDBP* ^G295S/G295S^ were generated from HPS4290. In this report, HPS1006 and HPS4290 are referred to as HC1 and HC2, respectively.

### 2.2 hiPSCs maintenance

hiPSCs were maintained in mTeSR1 medium (STEMCELL Technologies, Vancouver, Canada). When cells became 80% confluency, the hiPSCs were detached by ReLeSR (STEMCELL Technologies, Vancouver, Canada) according to manufacture protocol. The cramps of hiPSCs were seeded in another Matrigel-coated six-well plate. Cultural media were changed every day.

### 2.3 Differentiation of hiPSCs into EECM-BMEC-like cells and smooth muscle-like cells (SMLCs)

The extended endothelial cell culture method (EECM) was employed as an iPSCs-derived human *in vitro* BMEC model as previously described (Nishihara et al., 2020; Nishihara et al., 2021; Matsuo et al., 2023) (Figure 1C). CD31^+^ endothelial progenitor cells were generated by stimulating with CHIR99021 which activates Wnt/β-catenin signaling. To obtain a higher purity of CD31^+^ cells, the seeding density of hiPSCs on day -3 was optimized for each hiPSC clone with a range of 24,000–53,000/cm^2^ (Supplementary Table S1). After purifying the CD31^+^ cells using magnetic activated cell sorting (MACS), the cells were seeded on a collagen-coated 6-well plate. After 3–5 days of cultivating these cells, a small population of SMLCs is interspersed among the endothelial cells. When these cells are subjected to Accutase (Sigma-Aldrich, St. Louis, United States) treatment, the endothelial cells detach preferentially due to the differential detachability between endothelial and SMLCs. This difference can be exploited to selectively collect the initially detached endothelial cells. By repeating this process 2–3 times, a pure population of endothelial cells can be obtained and termed EECM-BMEC-like cells. EECM-BMEC-like cells from passages three to six were used for the following assays. The SMLCs remaining in the 6-well plate are left in the hECSR medium (Supplementary Table S2), and the conditioned medium is collected every 2–3 days when the medium changes, then filtered through a 0.22 μm filter and stored as previously described (Nishihara et al., 2021; Matsuo et al., 2023).

### 2.4 Permeability assay

Permeability of the EECM-BMEC-like cell monolayer was measured as previously described (Nishihara et al., 2021). In brief, EECM-BMEC-like cells were seeded onto 0.4 μm pore Transwell filter inserts (Corning 3,401, 12 wells 0.4 μm pore size, PC membrane, Corning, United States) with a consistent density of 1.12 × 10^5^ cells per 500 μL and cultured for 6 days. On day 6, the monolayers’ permeability was evaluated by determining the sodium fluorescein (NaFl, 376.3 Da, Sigma-Aldrich, St. Louis, United States) clearance. A10 μM concentration of NaFl was introduced to the top section of the Transwell inserts. Samples with the diffused fluorescent tracer were collected from the lower chamber every 15 min for up to 1 h. The fluorescence intensity was measured using a FlexStation 3 multi-well reader (Molecular Devices, San Jose, United States), with the excitation set at 485 nm and the emission detected at 530 nm. Each condition underwent the tests in triplicate. After each assay, the filters were stained as described below to confirm that the EECM-BMEC-like cell monolayer did not have holes that could result in incorrect permeability.

### 2.5 BMEC-like cell monolayer impedance measurement

An xCELLigence RTCA device (Agilent, St. Clara, United States) was used for real-time, label-free measurement of barrier integrity in 16-well plates with integrated gold electrodes (E-plate16: Agilent, St. Clara, United States). The plates were coated with collagen type Ⅳ. The cell-free background was measured in a culture medium for each well. Subsequently, EECM-BMEC-like cells were seeded at a density of 1 × 10^4^ cells/well and grown to confluence. The impedance of the monolayers was measured at 10 kHz every 15 min for 1 day until the impedance reached a plateau. Then, 4 μM CHIR99021 or DMSO as a control was added to the confluent monolayers, and the impedance was measured for another 2–3 days. Treatment or control wells were at least triplicate in each assay and assay were repeated at least two times. The output of the impedance measurements is the cell index, a dimensionless parameter that correlates with the strength of cell-cell contacts and cell adhesion. The cell-free background impedance is subtracted from the current impedance for each well to calculate the cell index. The values of the cell index were normalized to the last time point prior to treatment.

### 2.6 Flow cytometry analysis for cell surface expression of adhesion molecules

Cell surface expression of endothelial adhesion molecules was investigated as previously described (Nishihara et al., 2021). In this model, SMLC-derived conditioned media are required for VCAM-1 upregulation on the surface of BMEC-like cells (Nishihara et al., 2020). EECM-BMEC-like cells were cultured on 48-well plates with conditioned medium derived from SMLCs of the same clone in the presence or absence of stimulation with 1 ng/mL recombinant human TNF-α (210TA, R&D Systems, Minneapolis, United States) and 20 IU/mL recombinant human IFN-γ (285IF, R&D Systems, Minneapolis, United States) for 16 h at 37°C, 5% CO_2_. Stainings of cell surface adhesion molecules were detected using Attune NxT Flow Cytometer (Thermo Fisher Scientific, Waltham, United States). Mean fluorescence intensities were calculated by using FlowJo Ver10.9 (BD, Franklin Lakes, United States).

### 2.7 Immunofluorescence stainings

EECM-BMEC-like cell monolayers on the filter insert membranes, collagen type Ⅳ coated 8-well chamber plate or E-plate16 were analyzed for tight junction proteins as previously described (Nishihara et al., 2021). Briefly, cells were fixed with cold methanol (−20°C) for 20 s for claudin-5, occludin and VE-cadherin staining and with 4% paraformaldehyde for 10 min for double staining for TDP-43 and VE-cadherin. Then, cells were blocked and permeabilized with a solution of 5% skimmed milk and 0.1% Triton X-100, followed by incubation with primary and secondary antibodies (indicated in Supplementary Table S3). The nuclei were stained with 4′,6-diamidino-2-phenylindole (DAPI) at a concentration of 1 μg/mL. After DPBS washes, they were mounted with Mowiol (Sigma-Aldrich, St. Louis, United States). Images were captured using a BZ-X810 microscope (KEYENCE, Osaka, Japan). For TDP-43 mislocalization counting, immunofluorescence images were taken at 40x with reference to the DAPI channel alone to achieve similar cell density without bias. Ten pictures were taken in each clones. The number of mislocalizations in the cytoplasm was counted by three different examiners independently in a blinded fashion to eliminate their bias.

### 2.8 Quantitative-RT PCR

The total RNA was extracted from confluent EECM-BMEC-like cells using an RNA extraction kit (Roche, Switzerland). Total RNA was reverse transcribed using AffinityScript cDNA Synthesis Kit (Agilent, St. Clara, United States), and amplification was performed on a CFX384 Touch Real-Time PCR Detection System (BioRad) using Brilliant Ⅲ Ultra-Fast SYBER Green Master Mixes (Agilent, St. Clara, United States). Primer sequences are shown in Supplementary Table S4. The relative expression of each mRNA was calculated by the comparative threshold cycle method and normalized to β-actin mRNA expression.

### 2.9 Activation of the Wnt/β-catenin signaling pathway in EECM-BMEC-like cells

Glycogen synthase kinase-3 (GSK-3) inhibitor, CHIR99021 was used as a Wnt/β-catenin pathway activator. EECM-BMEC-like cells at passage four or five were thawed as previously described (Nishihara et al., 2021). EECM-BMEC-like cells were treated with 4 μM CHIR99021 or DMSO as a control in hECSR medium until usage. EECM-BMEC-like cells were seeded onto Transwell filters, chamber slides, or plates for permeability assays, immunocytochemistry, or adhesion molecule phenotyping as described above.

### 2.10 Immune cell adhesion assay under static conditions on a 96-well plate

EECM-BMEC-like cells are seeded onto 10 μg/mL collagen type Ⅳ-coated 96-well plates (Coning, Coning, United States) at a density of 40,000 cells per well. The following day, the medium was replaced with a conditioned medium of SMLCs derived from the same hiPSC clone and incubated for 16 h in the presence or absence of 0.1 ng/mL TNF-α and 2 IU/mL IFN-γ. On the day of the experiment, fluorescently-labeled PBMCs were prepared and adhesion assays were performed using a previously described method (Nishihara et al., 2021), with slight modifications. Briefly, PBMCs were isolated from healthy donors using BD Vacutainer CPT Mononuclear Cell Preparation Tube (BD, Franklin Lakes, United States) as manufacture protocol. Isolated PBMCs were frozen until use. PBMCs from allogeneic healthy donors were thawed on the day of experiments using T-cell wash buffer (Supplementary Table S2) and were centrifuged (280 g, 5 min, 20°C–25°C) to remove the preservation reagent. PBMCs were incubated with the T-cell medium (Supplementary Table S2) containing 1 nM CMFDA dye (Invitrogen, Waltham, United States) for 30 min at 37°C, 5% CO_2_. Unconjugated fluorophores were washed out and labeled PBMCs were resuspended in the T-cell medium. Ficoll-Paque PLUS (Sigma-Aldrich, St. Louis, United States) was carefully added underneath the cell suspension and the tube was centrifuged (770 g, 20 min at 20°C–25°C, minimum brake and acceleration) and live PBMCs existed in the interphase between the buffer and the Ficoll-Paque were collected and resuspended with the migration assay medium (Supplementary Table S2) at a concentration of 2.0 × 10^5^/mL. 100 μL of PBMC suspension (2.0 × 10^4^/well) was evenly added using electronic multi-dispenser pipettes (Eppendorf, Hamburg, Germany) to each well of a 96-well plate on top of EECM-BMEC-like cells. The plate was placed on a plate shaker under light-shielded conditions. After 15 min, the plate was rotated 90 degrees and incubated for another 15 min on the shaker. After sucking up the unattached cells, 200 μL of DPBS (Thermo Fisher Scientific, Waltham, United States) was gently added to each well using electronic multi-dispenser pipettes for washing. Aspirated DPBS and repeated washing wells one more time and then fixed cells with 50 μL cold methanol (−20°C) for 1 min. After washing the wells with 100 μL of DPBS, 100 μL of Mowiol was added to each well. Images of the wells were captured using a BZ-X810 microscope (KEYENCE, Osaka, Japan) at a magnification of ×10. To mitigate the potential for selection bias regarding the photograph’s location, the setup was calibrated to ensure automatic capture of each well’s center. Before analyzing the number of adhered PBMCs, phase contrast images were used to exclude wells in which a part of the EECM-BMEC-like cell monolayer was detached. The number of adherent CMFDA-labeled PBMCs was automatically counted in each captured image using the hybrid cell count software BZ-X analyzer in each visual field (KEYENCE, Osaka, Japan).

### 2.11 Statistical analysis

GraphPad Prism seven software (GraphPad Software, La Jolla, United States) was used for statistical analyses that included degree of freedom calculations. The displayed data is the mean ± SD. An unpaired *t*-test was used to determine statistical significance when comparing two groups. The corresponding figure legends provide information on the specific statistical methods applied to each experiment.

### 2.12 Study approval

The study was approved by the ethics committees of the Medical Faculties of Yamaguchi University (number H2021-133-3) and Keio University School of Medicine Ethics Committee (approval no.20080016).

## 3 Results

### 3.1 Establishment and characterization of EECM-BMEC-like cells and SMLCs

We first differentiated EECM-BMEC-like cells and SMLCs to establish *in vitro* BMEC and smooth muscle cell models from an ALS patient. One hiPSC clone from an ALS patient carrying *TARDBP* ^N345K/+^ and two healthy controls (HC1, HC2) were used in the study. In addition, by introducing different *TARDBP* mutations into HC2, two distinct *TARDBP* mutant iPSC clones (*TARDBP* ^G295S/G295S^ and *TARDBP* ^K263E/K263E^) were generated on the same genetic background (Figure 1A). The HC- and ALS-derived iPSCs were differentiated BMEC-like cells and SMLCs using EECM (Figure 1C). Both HC- and ALS-derived hiPSCs can be differentiated into EECM-BMEC-like cells, which resemble primary human BMECs in spindle-shaped morphology (Figure 1D). We further confirmed the continuous adherens junction in the staining for VE-cadherin and the intercellular localization of tight junction proteins such as claudin-5 and occludin (Figure 1D). SMLCs derived from each hiPSC clone also confirmed characteristics of smooth muscle cells by staining such as alpha-smooth muscle actin (αSMA), smooth muscle protein 22-alpha (SM22α), and calponin (Figure 1D). Differentiations of each clone were performed at least 5 times, and their reproducibility was confirmed. These data indicate that both HC- and ALS-derived hiPSCs successfully differentiated into EECM-BMEC-like cells and SMLCs as previously reported (Nishihara et al., 2020) and used further studies for diffusion barrier functions and immune cell interaction with BMEC-like cells.

### 3.2 ALS patient (*TARDBP* ^N345K/+^)-derived EECM-BMEC-like cells showed loss of tight junctions

**FIGURE 2:**
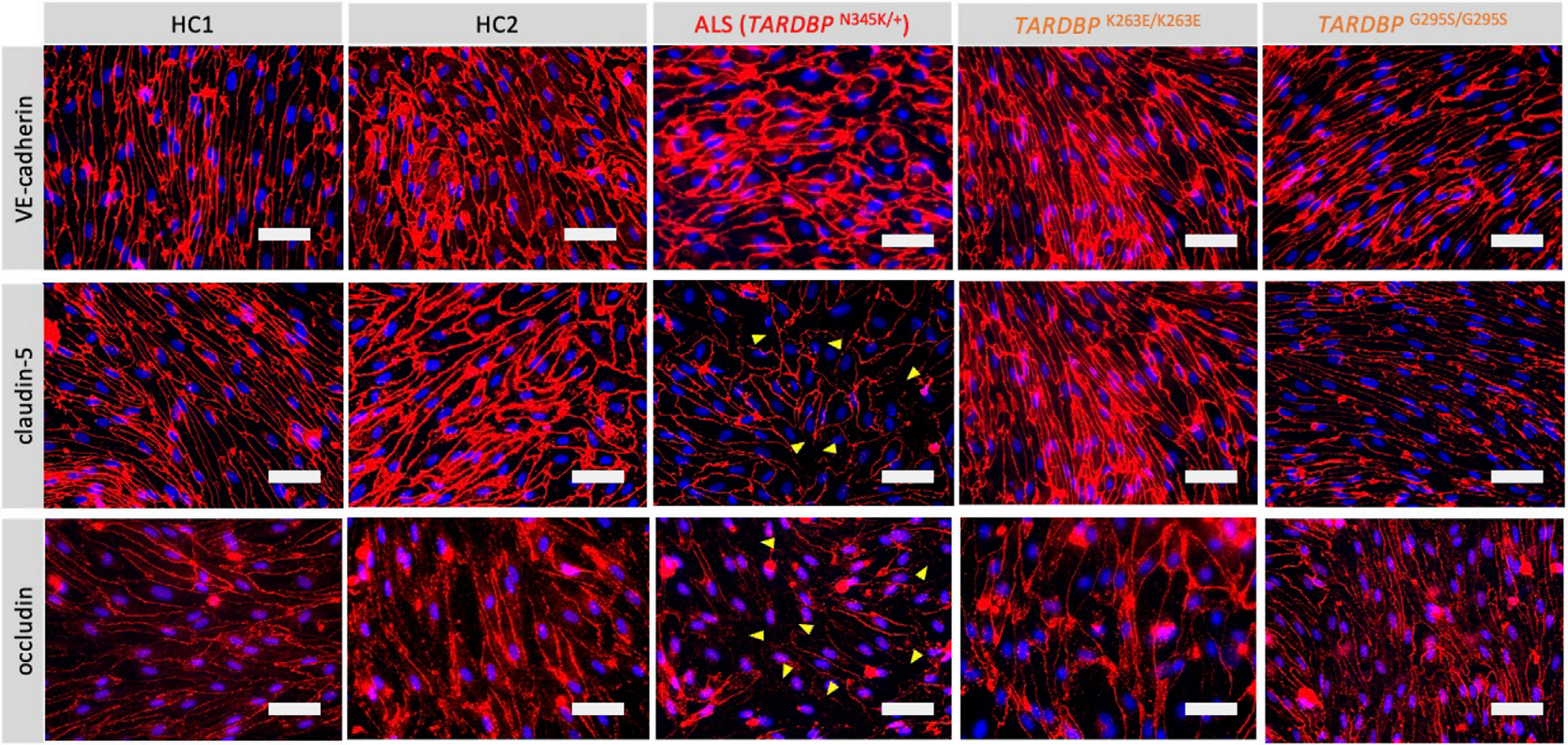
ALS patient-derived EECM-BMEC-like cells exhibit tight junction discontinuation. EECM-BMEC-like cells were cultivated onto a 0.4 um pore size Transwell filter for 6 days. Junctions were stained for VE-cadherin, claudin-5, or occludin (red), and nuclei were stained with DAPI (blue). Yellow arrowheads indicate visible discontinuations of the claudin-5 and occludin. Each staining represents results from at least five independent experiments, each performed with five separate differentiation batches. Scale bar = 50 μm.

Since autopsy samples from the brains or spinal cords of ALS patients showed decreased expression levels of tight junction proteins (Garbuzova-Davis et al., 2012), we next examined if ALS patient-derived EECM-BMEC-like cells recapitulate disrupted tight junction. We compared the morphology and the staining of adherence and tight junctions of ALS-derived EECM-BMEC-like cells to HCs-derived cells. As a result, ALS patient (*TARDBP* ^N345K/+^)-derived EECM-BMEC-like cells had a larger morphology despite the same seeding density (Figure 2). There was no significant difference in the staining of VE-cadherin, the adherens junction, between ALS patient (*TARDBP* ^N345K/+^)-derived EECM-BMEC-like cells and HCs-derived cells. Interestingly, we found that ALS patient (*TARDBP* ^N345K/+^)-derived cells showed discontinuation of tight junction proteins claudin-5 and occludin, which was not observed in HCs-derived cells. Next, we asked whether *TARDBP* ^G295S/G295S^ and *TARDBP* ^K263E/K263E^-derived cells, which are single *TARDBP* mutant models, recapitulate the disrupted tight junctions. Contrary to expectations, *TARDBP* ^G295S/G295S^ and *TARDBP* ^K263E/K263E^ -derived EECM-BMEC-like cells showed continuous tight junctions comparable to HC-derived cells (Figure 2). These results indicate that *TARDBP* ^N345K/+^ EECM-BMEC-like cells mimic the tight junction discontinuation observed in ALS patients’ autopsied CNS samples, however, the *TARDBP* gene-edited model (*TARDBP* ^K263E/K263E^ and *TARDBP* ^G295S/G295S^) did not recapitulate the disrupted tight junctions.

### 3.3 ALS patient (*TARDBP* ^N345K/+^)-derived EECM-BMEC-like cells showed impaired diffusion barrier properties

**FIGURE 3:**
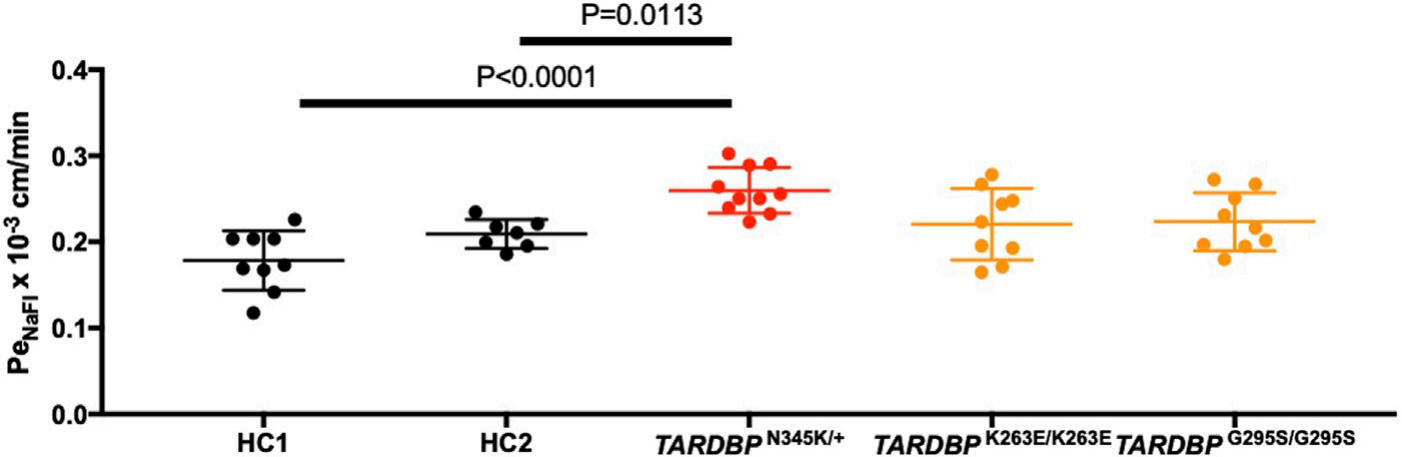
EECM-BMEC-like cells derived from ALS patients exhibited impaired diffusion barrier properties. EECM-BMEC-like cells derived from HCs (black), ALS clone (red), or gene-editing clones (orange) were grown on 0.4 μm pore size Transwell filters for 6 days to form a confluent monolayer in monoculture. Permeability was measured on day 6. The permeability was calculated from the fluorescence intensity of 0.37 kDa sodium fluorescein (NaFl) across the EECM-BMEC-like cell monolayers. Each symbol showed the permeability of independent filters with at least three independent differentiations, each performed in at least triplicate. Data were shown as mean ± SD. Statistical analysis: unpaired *t*-test. P-values are indicated in the corresponding figures.

The leakage of blood proteins such as IgG and fibrin from blood vessels has been reported in autopsied brains and spinal cords of ALS patients (Winkler et al., 2013). Still, it was not clear whether this leakage was a result of the end-stage neurodegeneration and *postmortem* changes or indicated a primary abnormality in the BMECs. To answer this question, we asked if ALS patient-derived *in vitro* EECM-BMEC-like cells show increased permeability in the absence of neurodegeneration or inflammation. Comparing permeability for small molecule tracers, as a marker for diffusion barrier properties, ALS patient (*TARDBP* ^N345K/+^)-derived EECM-BMEC-like cells showed significantly increased NaFl permeability compared to HCs (Figure 3). Next, we asked whether the specific *TARDBP* mutations (*TARDBP* ^K263E/K263E^ and *TARDBP* ^G295S/G295S^) contribute to impaired diffusion barrier properties. The permeability of *TARDBP* ^K263E/K263E^ and *TARDBP* ^G295S/G295S^-derived EECM-BMEC-like cells was not significantly increased compared to isogenic control (HC2)-derived cells (Figure 3). These data indicate that EECM-BMEC-like cells derived from an ALS patient (*TARDBP* ^N345K/+^) exhibit impaired junctional integrity and this discontinuation of tight junctions (Figure 2) has functional significance as reflected in the higher permeability of small molecules. Impotently this impaired diffusion barrier properties were demonstrated in the absence of the factor of neurodegeneration and neuroinflammation.

### 3.4 ALS patient (*TARDBP* ^N345K/+^)-derived EECM-BMEC-like cells exhibited increased expression of cell surface adhesion molecules

**FIGURE 4:**
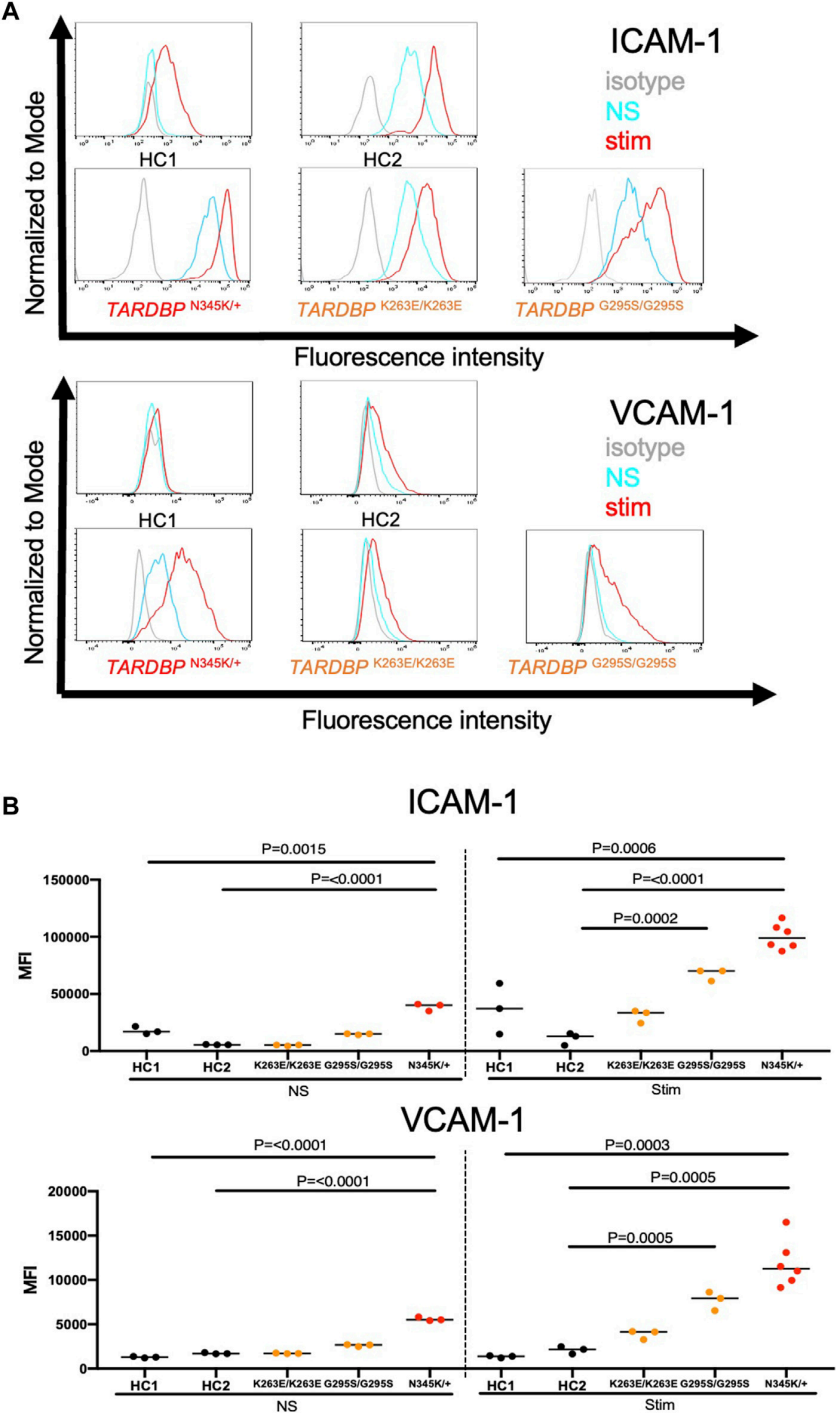
ALS patient-derived EECM-BMEC-like cells showed enhanced endothelial adhesion molecules. EECM-BMEC-like cells were seeded onto type Ⅳ collagen-coated well plates and treated with conditioned medium from the same hiPSC clone, with or without proinflammatory cytokine stimulation (1 ng/mL TNF-α + 20 IU/mL IFN-γ). A flow cytometer was used to detect cell surface immunostaining of EECM-BMEC-like cells for the adhesion molecules ICAM-1 and VCAM-1. Gray, blue, and red lines indicate isotype control, non-stimulated (NS), and 16-h proinflammatory cytokine-stimulated conditions, respectively. **(A)** Representative histograms for ICAM-1 and VCAM-1 were shown for each clone. At least three replicates were analyzed for each clone. **(B)** The change in the geometric mean of cell surface ICAM-1 and VCAM-1 of EECM-BMEC-like cells was analyzed by flow cytometer. Each symbol represents an experiment using an independently differentiated sample. left side: non-stimulated condition (NS), right side: stimulated condition with 1 ng/mL TNF-α and 20 IU/mL IFN-γ(Stim). Statistical analysis: unpaired *t*-test. P-values were indicated in the corresponding figures.

ALS-patient autopsy samples show immune cell infiltrations into the CNS (Henkel et al., 2004; Garofalo et al., 2022) as another vascular barrier-disrupting feature. Therefore, we next checked if ALS patient-derived EECM-BMEC-like cells possess important endothelial adhesion molecules ICAM-1 and VCAM-1, which controls immune cell infiltration into the CNS (Marchetti and Engelhardt, 2020). We examined the expression of cell surface endothelial adhesion molecules in conditions with and without proinflammatory cytokine stimulation, as a representative of physiological and peripheral inflammation-stimulated conditions, respectively. When culturing EECM-BMEC-like cells in an SMLC-conditioned medium, ALS patient (*TARDBP* ^N345K/+^) -derived EECM-BMEC-like cells expressed cell surface ICAM-1 under nonstimulated conditions, which is enhanced upon proinflammatory cytokine stimulation (Figure 4A). Cell surface VCAM-1 expression is minimal without cytokine stimulation but is effectively induced upon proinflammatory cytokine stimulation in the presence of SMLC-conditioned medium, as previously described (Nishihara et al., 2020) (Figure 4A). We next compared cell surface adhesion molecule expression in ALS-derived EECM-BMEC-like to HCs-derived cells. We found that ALS patient (*TARDBP* ^N345K/+^)-derived EECM-BMEC-like cells expressed increased ICAM-1 and VCAM-1 under both unstimulated and stimulated conditions compared to HCs (Figures 4A, B). Next, we asked if gene-edited *TARDBP* ^K263E/K263E^ and *TARDBP* ^G295S/G295S^-derived EECM-BMEC-like cells showed increased expression of endothelial adhesion molecules in both unstimulated and stimulated conditions compared to their isogenic control (HC2). As a result, *TARDBP* ^G295S/G295S^-derived cells showed enhanced expression of cell surface ICAM-1 and VCAM-1 in cytokine-stimulated conditions but *TARDBP* ^K263E/K263E^-derived cells didn’t show enhanced adhesion molecule expression. These data suggest that ALS patient (*TARDBP* ^N345K/+^)-derived EECM-BMEC-like cells express enhanced ICAM-1 and VCAM-1, which may contribute to immune cell trafficking into the CNS. The gene-editing *TARDBP* mutations (*TARDBP* ^K263E/K263E^ and *TARDBP* ^G295S/G295S^) didn’t recapitulate this phenotype completely.

### 3.5 ALS patient (*TARDBP* ^N345K/+^)-derived EECM-BMEC-like cells enhance PBMC adhesion

**FIGURE 5:**
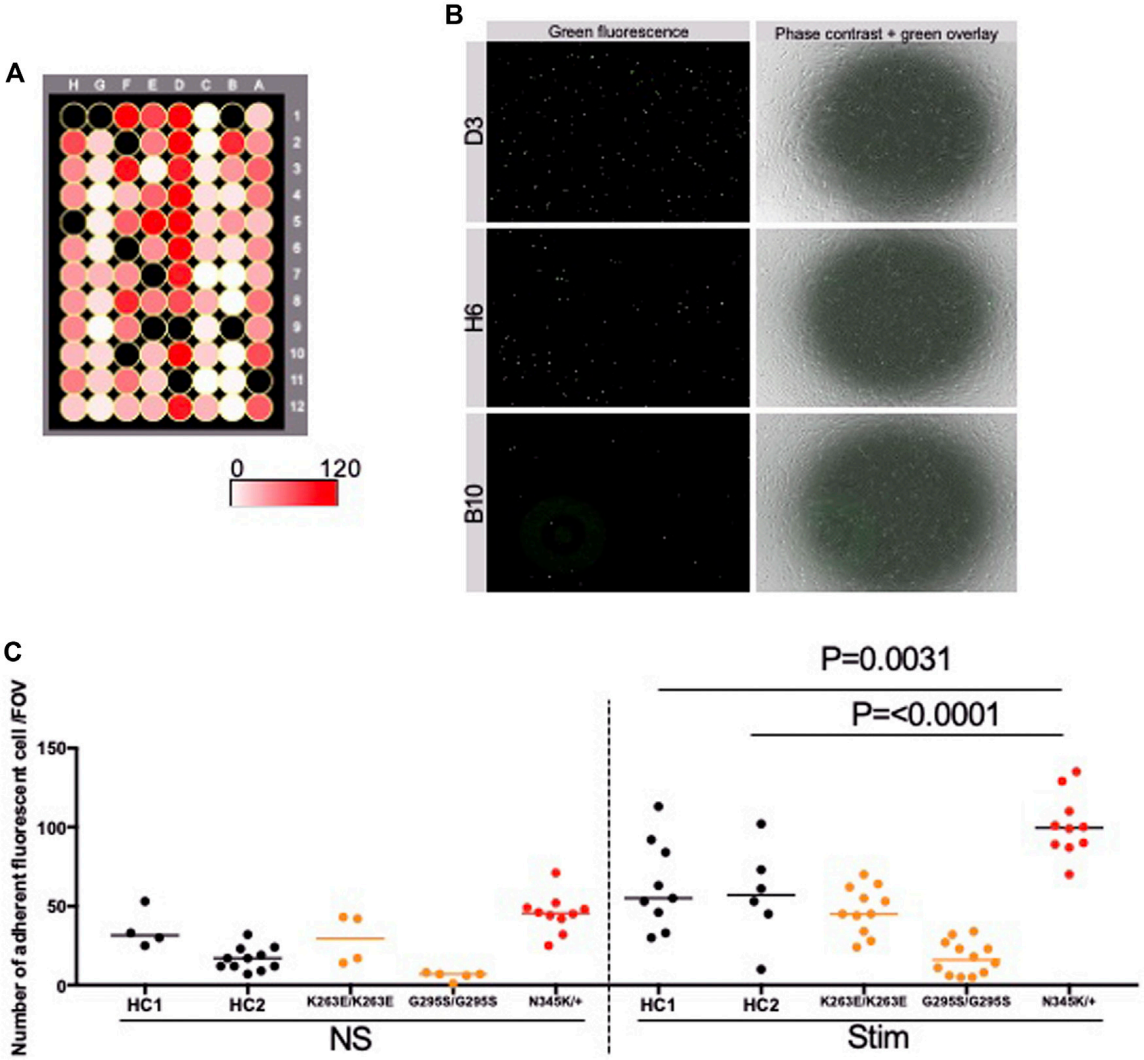
ALS patient-derived EECM-BMEC-like cells enhanced PBMCs adherent EECM-BMEC-like cells were seeded onto type Ⅳ collagen-coated 96-well plates and treated with conditioned medium from the same hiPSC clone, with or without 16 h proinflammatory cytokines stimulation (0.1 ng/mL TNF-α + 2 IU/mL IFN-γ). Fluorescently labeled PBMCs from allogeneic healthy donors interacted with the EECM-BMEC-like cell monolayer for 30 min. After washing, phase contrast and fluorescence images from the center of each well were photographed automatically. The number of adherent PBMCs was automatically counted using the hybrid cell count software BZ-X analyzer. **(A)** The heat map of the number of adherent PBMCs in the wells. A1-12 were *TARDBP* ^K263E/K263E^-derived clone under non-stimulated conditions. B1-6 were *TARDBP* ^K263E/K263E^-derived clone under cytokine-stimulated conditions. B7-12 were *TARDBP* ^G295S/G295S^-derived clone under non-stimulated conditions. C1-12 were *TARDBP* ^G295S/G295S^-derived clone under cytokine-stimulated conditions. D1-12 were ALS patient (*TARDBP* ^N345K/+^)-derived clone under cytokine-stimulated conditions. E1-6 were ALS patient (*TARDBP* ^N345K/+^)-derived clone under non-stimulated conditions. E7-12 were HC1 under non-stimulated conditions. F1-12 were HC1 under cytokine-stimulated conditions. G1-12 were HC2 under cytokine-stimulated conditions. H1-12 were HC2 under cytokine-stimulated conditions. Black-colored wells were excluded because the cell layers in the center of the wells were detached. **(B)** Representative images of each well. Green fluorophore-labeled adherent PBMCs and monolayers of EECM-BMEC-like cells on phase contract image. D3, H6, and B10 were the locations of the wells representing a high, middle, and low number of adherent PBMCs, respectively. **(C)** Number of adherent PBMCs onto EECM-BMEC-like cells derived from each clone in the presence or absence of proinflammatory cytokines stimulation. A dot represented the number of adherent PBMCs in the FOV at the center of each well. Left side: non-stimulated condition (NS), right side: stimulated condition with 0.1 ng/mL TNF-α and 2 IU/mL IFN-γ (Stim). Data are shown as mean ± SD. The Statistical analysis: unpaired *t*-test. *P*-values were indicated in the corresponding figures.

We further investigated whether the enhanced adhesion molecules on the surface of *TARDBP* ^N345K/+^-derived EECM-BMEC-like cells are functional and are correlated to increased immune cell interaction with endothelium. To this end, the immune cell adhesion assay was performed and the numbers of adherent allogeneic PBMCs on EECM-BMEC-like cells monolayer from each clone were compared. This assay was done based on the previously reported methodology (Nishihara et al., 2021) with modification. EECM-BMEC-like cells from all clones were seeded in a 96-well plate and assayed in the same setup to minimize the variability between the experiments (Figure 5A). To avoid selection bias, the center of all wells was automatically photographed. Although the cells in all wells formed a complete monolayer before the assay, some of the cells were detached after the assay due to the fixation and washing process, therefore, we excluded these wells from the analysis using phase contrast images. The number of adherent PBMCs was then counted in the same setup concerning the fluorescence threshold for image analysis using the hybrid cell count software BZ-X analyzer. The fluorescently labeled PBMCs dispersed well and adhered to the monolayer of EECM-BMEC-like cells in each well (Figure 5B). When we compared the number of adherent PBMCs, ALS patient (*TARDBP* ^N345K/+^)-derived EECM-BMEC-like cells showed a significantly increased number of adherent PBMCs under proinflammatory cytokine-stimulated conditions compared to HCs (Figure 5C). In contrast, *TARDBP* ^K263E/K263E^ and *TARDBP* ^G295S/G295S^-derived EECM-BMEC-like cells didn't show such enhanced PBMCs attachment compared to HC2. Thus, the elevated levels of cell surface ICAM-1 and VCAM-1 in EECM-BMEC-like cells derived from the ALS patient (*TARDBP* ^N345K/+^) were functional in adhering more immune cells. Furthermore, this enhanced PBMC adhesion to the ALS patient-derived EECM-BMEC-like cells happened independent of changes within the CNS including neurodegeneration.

### 3.6 ALS patient (*TARDBP* ^N345K/+^)-derived EECM-BMEC-like cells showed mislocalization of TDP-43, and activation of Wnt/β-catenin signaling restored endothelial dysfunctions

**FIGURE 6:**
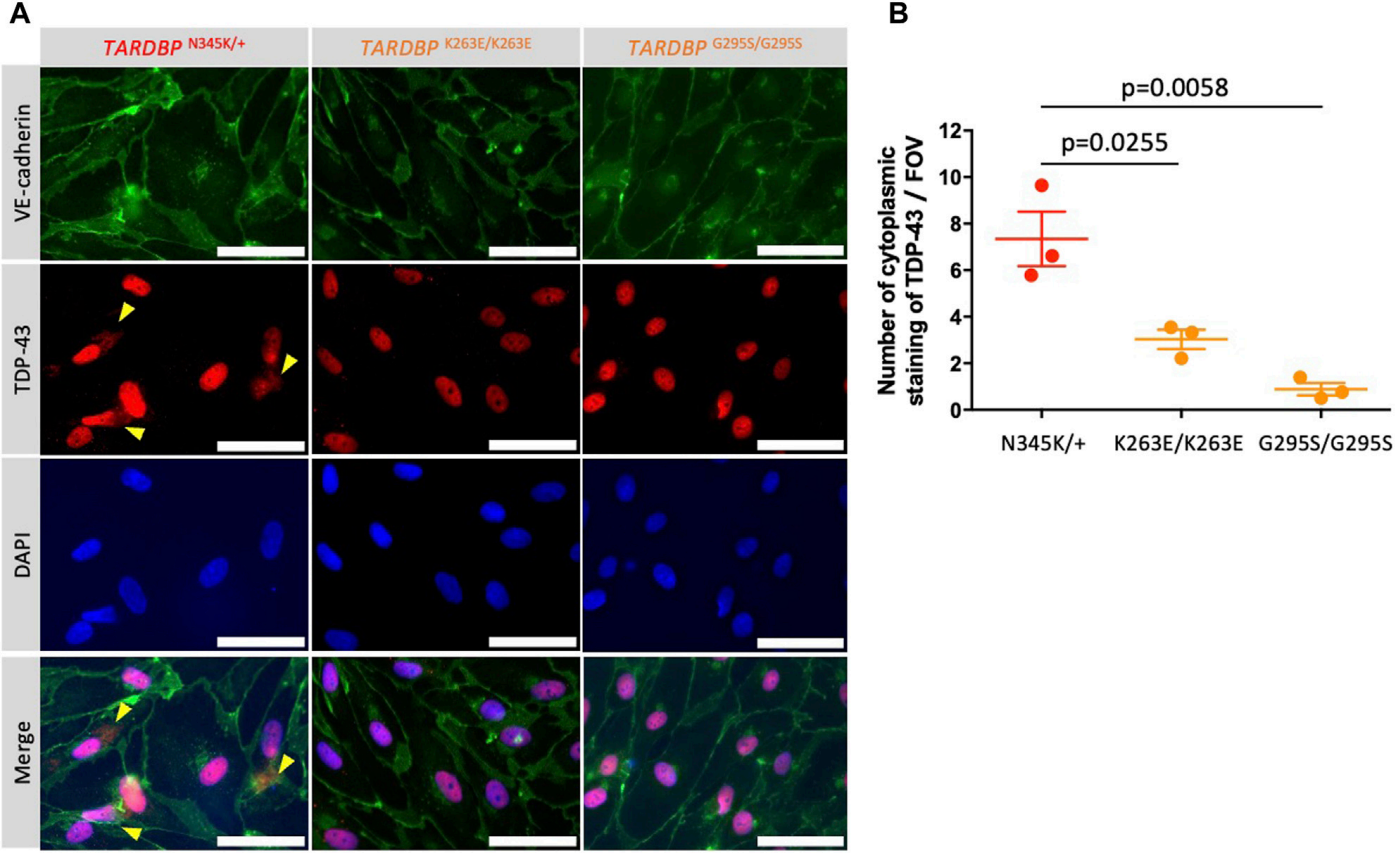
ALS patient-derived EECM-BMEC-like cells showed cytoplasmic mislocalization of TDP-43. **(A)** Representative images of triple staining TDP-43 (red), VE-cadherin (green), and DAPI (blue). TDP-43 proteins colocalized in nuclei were stained purple, whereas TDP-43 proteins mislocalized in the cytoplasm remained red (yellow arrow). Each staining represents results from at least three independent experiments, each performed with three separate differentiation batches. Scale bar = 30 μm **(B)** TDP-43 staining in the cytoplasm were counted in a field of view of 40x images. Each dot represents the mean of the counts made by three independent examiners, each investigating 10 pictures. The Statistical analysis: unpaired *t*-test. P-values are indicated in the corresponding figures.

Autopsy samples show cytoplasmic aggregates of TDP-43 not only in neurons and glial cells but also in brain endothelial cells in ALS patients (Ferrer et al., 2021). The staining of TDP-43 in the cytoplasm indicates the mislocalization of nuclear TDP-43, which leads to the impairment of TDP-43 functions in the nuclei. In addition, mouse data show that mislocalization of TDP-43 leads to tight junction disruption, resulting in a leaky barrier. Therefore, we examined whether our model recapitulates these morphological changes. TDP-43 localization was assessed by co-staining with DAPI and VE-cadherin. Indeed, increased cytoplasmic stainings of TDP-43 were found in patient-derived (*TARDBP* ^N345K/+^) but not gene-edited clones (*TARDBP* ^K263E/K263E^ and *TARDBP* ^G295S/G295S^) and HC (Figure 6A; Supplementary Figure S1). The number of cytoplasmic staining in a field of view was significantly higher in the ALS patient (*TARDBP* ^N345K/+^)-derived EECM-BMEC-like cells compared to gene-edited cells (Figure 6B).

**FIGURE 7:**
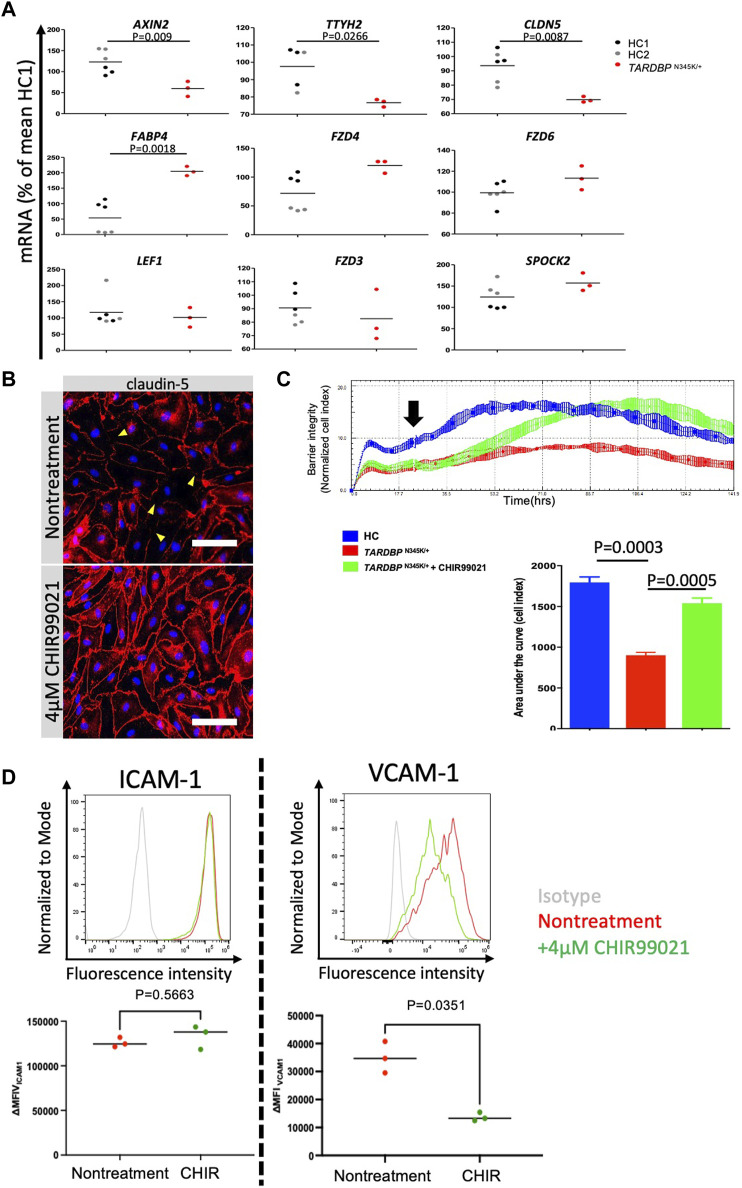
Activation of downregulated Wnt/beta-catenin signaling restored impaired barrier properties in ALS patient-derived EECM-BMEC-like cells. **(A)** *AXIN2*, *TTYH2*, *CLDN5*, *LEF1*, FZD3, *FZD4*, *FZD6, SPOCK2, and FABP4* mRNA expression was measured via quantitative real-time polymerase chain reaction (RT-qPCR) in HC1, HC2, and ALS patient (*TARDBP* ^N345K/+^)-derived EECM-BMEC-like cells. The mean value of the HC1-derived sample was set to 100% and used as a normalization factor for each value. Each dot represents one sample, and qPCR was performed at least three times for each sample. The black bar represented the mean of mRNA expression. **(B)** ALS-patient (*TARDBP* ^N345K/+^)-derived EECM-BMEC-like cells were cultivated onto collagen type Ⅳ coated plate for 3 days in the presence of 4 μM CHIR99021. Junctions were stained for claudin-5 (red), and nuclei were stained with DAPI (blue). Each staining represents results from at least three independent experiments, each performed with three separate differentiation batches. Scale bar = 40 μm **(C)** Left: The impedance of monolayered EECM-BMEC-like cells was shown. Cells derived from HC1 (blue) or ALS patient (*TARDBP* ^N345K/+^) (red) were seeded on E-plate 16. Some wells were treated with 4 μM CHIR99021 (green). The black arrow indicates the timing when CHIR99021 or DMSO control was added to each well. Each condition had at least three replicates. Higher normalized cell index values indicate increased barrier integrity. Right: Area under the curve of the cell index in each condition. **(D)** The images showed representative ICAM-1 and VCAM-1 expression histograms of *TARDBP* ^N345K/+^ carrying ALS patient-derived EECM-BMEC-like cells in the presence or absence of CHIR99021 treatment. EECM-BMEC-like cells were treated with conditioned medium from the same hiPSC clone-derived SMLCs with proinflammatory cytokine stimulation (1 ng/mL TNF-α + 20 IU/mL IFN-γ) in the presence or absence of 4 μM CHIR99021 treatment. The expression of ICAM-1 or VCAM-1 was detected by flow cytometry analysis. Each staining represented results from three independent experiments. The below dot graph showed the change in the mean (ΔMFI = MFI staining–MFI isotype) of cell surface ICAM-1 and VCAM-1 of *TARDBP* ^N345K/+^ carrying ALS patient-derived EECM BMEC-like cells. Each symbol represented an experiment using an independently differentiated sample. Bars showed the mean ΔMFI value of three experiments. Statistical analysis: unpaired *t*-test. *P*-values are indicated in the corresponding figures.

Having established that ALS patient (*TARDBP* ^N345K/+^)-derived EECM-BMEC-like cells are suitable for modeling endothelial dysfunction, we next explored whether this model is appropriate for evaluating therapeutic strategies targeting the endothelial cells in the treatment of ALS. The canonical Wnt/β-catenin signaling pathway is a well-known signaling pathway involved in developing and maintaining the BBB (Stenman et al., 2008; Daneman et al., 2009; Zhou et al., 2014). In addition, several reports have shown a functional link between TDP-43 and canonical Wnt/β-catenin signaling in brain endothelial cells of mouse models (Omar et al., 2023; Arribas et al., 2024). In fact, we found that ALS patient (*TARDBP* ^N345K/+^)-derived EECM-BMEC-like cells showed significantly decreased expressions of *AXIN2*, *CLDN5*, and *TTYH2*, which were reported as the canonical Wnt-target genes (Gastfriend et al., 2021b; Arribas et al., 2024) (Figure 7A). Additionally, the fatty acid binding protein encoding *FABP4*, which has been reported to be downregulated by Wnt/β-catenin signaling activation (Gastfriend et al., 2021b) and is depleted in CNS vascular endothelial cells (Sabbagh et al., 2018) was significantly upregulated in ALS patient (*TARDBP* ^N345K/+^)-derived EECM-BMEC-like cells. These results motivate us to investigate if activation of Wnt/β-catenin signaling could rescue the phenotype of ALS patient (*TARDBP* ^N345K/+^)-derived EECM-BMEC-like cells. Consequently, a GSK-3 inhibitor, CHIR99021, a chemical activator of Wnt/β-catenin signaling, restored the disrupted claudin-5 staining in these cells (Figure 7B). Interestingly, the Wnt/β-catenin signaling activation increased the impedance of the monolayer of ALS patient (*TARDBP* ^N345K/+^)-derived EECM-BMEC-like cells to levels comparable to HC (Figure 7C). The gene-edited clones (*TARDBP* ^K263E/K263E^ and *TARDBP* ^G295S/G295S^) showed higher impedance, comparable to ALS patient (*TARDBP* ^N345K/+^)-derived cells, and CHIR99021 treatment did not further increase their impedance anymore (Supplementary Figure S2; Figure 7C). We confirmed this effect using another method involving the permeability for small molecule tracers NaFl. Wnt/β-catenin signaling activation also reduced the permeability of ALS patient (*TARDBP* ^N345K/+^)-derived EECM-BMEC-like cells to levels comparable to HC-derived cells (mean Pe _NaFl_ = 0.18 ± 0.017 × 10^-3^ cm/min versus Figure 3 HC1 or HC2 mean Pe _NaFl_ = 0.18 ± 0.03 × 10^−3^ or 0.21 ± 0.005 × 10^−3^ cm/min, respectively). Furthermore, Wnt/β-catenin activation suppressed the increased expression of VCAM-1 but not ICAM-1 on the surface of ALS patient (*TARDBP* ^N345K/+^)-derived EECM-BMEC-like cells (Figure 7D). These results suggest that ALS patient (*TARDBP* ^N345K/+^)-derived EECM-BMEC-like cells showed cytoplasmic mislocalization of TDP-43, which could lead Wnt/β-catenin downregulation. Indeed, activation of Wnt/β-catenin signaling in fully differentiated EECM-BMEC-like cells from the ALS patient (*TARDBP* ^N345K/+^), which showed mislocalization of TDP-43, rescued the impaired barrier properties. This highlights that damaged endothelial cells are repairable and can be targeted for therapy.

## 4 Discussion

In the current study, we applied our previously established hiPSC-derived BMEC-like cell differentiation method, EECM-BMEC-like cells, which is useful for studying the endothelial cell morphology and the interactions between the endothelial cells and immune cells, to the study of BMEC involvement in ALS pathogenesis. This application allowed us to establish a novel human ALS patient (*TARDBP* ^N345K/+^)-derived BMEC-like cells. To date, reports of BBB dysfunction in ALS patients have been limited to studies of postmortem autopsy specimens, making it unclear whether BBB dysfunction is the cause of neurodegeneration or merely a secondary consequence. However, several studies of the rodent models have demonstrated the BBB disruption preceding neuronal loss. For example, in G93A human *SOD1* transgenic mice (*SOD1* ^G93A^), which are widely used as animal models for ALS, motor neuron numbers decrease and neurological symptoms appear at 13 weeks of age. However, 1) the leakage of IgG and Prussian blue has already begun at 8 weeks when the number of motor neurons has not yet decreased (Zhong et al., 2008). 2) Increased expression of matrix metalloproteinase-9, a key extracellular matrix degradation enzyme, and decreased staining of tight junction and basement membrane components were already observed at 10 weeks (Miyazaki et al., 2011). 3) IgG deposition, increased ICAM-1, and activation of microglia in the spinal cord were observed from the asymptomatic period around day 40–80 (6–11 weeks) (Alexianu et al., 2001). 4) Even at the early stage of the disease (13 weeks), endothelial cells and endfeet of astrocytes are swollen and vacuolized in the brainstem and spinal cord (Garbuzova-Davis et al., 2007). Such “pre-symptomatic barrier dysfunction” has also been reported in *SOD1* ^G93A^ rats and *SOD1* ^G86R^ mice (Nicaise et al., 2009; Milane et al., 2010). Serum protein leakage and immune cell infiltration into the CNS have also been reported in *TARDBP*-related mouse models, although the studies did not focus on the onset of the alteration (Sasaki et al., 2015; Zamudio et al., 2020). This evidence encouraged us to investigate whether BBB functions are intrinsically altered in human ALS, independent of neurodegeneration, and contribute to disease onset or progression. The differences in BMEC constituent proteins between animal models and humans, along with the low success rate of translating results from animal models to human treatments (Uchida et al., 2011; Lecuyer et al., 2017; Song et al., 2020; Uchida et al., 2020), have motivated us to develop patient-derived models. We have successfully established a novel ALS patient (*TARDBP* ^N345K/+^)-derived BMEC-like cells, which recapitulates two major vascular barrier disruptive mechanisms, 1) increased permeability and 2) enhanced immune cell adhesion, and opens an avenue to investigate this challenging hypothesis.

In the present study, an ALS patient (*TARDBP* ^N345K/+^)-derived EECM-BMEC-like cell exhibited discontinuation of tight junctions resulting in increased permeability of small molecule tracers. This model also showed increased expression of ICAM-1 and VCAM-1, which are major adhesion molecules for promoting immune cell trafficking into the CNS. Moreover, enhanced endothelial adhesion molecules were functional since we showed a higher number of PBMCs adhered to ALS patient (*TARDBP* ^N345K/+^)-derived EECM-BMEC-like cells compared to HCs-derived cells. Indeed, the increased expression of ICAM-1 or VCAM-1 in *TARDBP* mutated endothelial cells has been reported (Hipke et al., 2023; Arribas et al., 2024). One of the advantages of disease modeling using hiPSCs is that we can simplify the model and investigate how a patient genetic background affects the specific cell type. In this study, we showed that dysfunction of BMEC-like cells occurs independently of the impact of degenerating neurons or glial cells. Similar to our result, another group reported on the impaired BMEC functions of hiPSC-derived BMEC-like cells from ALS patients harboring *SOD1* and *C9orf72* mutations (Katt et al., 2019). However, the association between ALS-related genes and adhesion molecules on the surface of BMEC has not been reported. This is because the differentiation method in the previous report is distinct (Lippmann et al., 2014), which does not properly recapitulate cell surface adhesion modules (Nishihara et al., 2020). In addition, these *SOD1* or *C9orf72* mutation-carrying hiPSC-derived BMEC-like cells showed a lower transendothelial electrical resistance (TEER) compared to HCs, but the permeability of the small molecule tracer was not significantly different compared to HCs. It is unclear whether the discrepancy in permeability results was due to differences in the mutation in different genes or to differences in the differentiation method. In any case, these data suggest that ALS-related gene mutations have an impact not only on neurons and glial cells, but also on other important physiological functions such as vascular barrier properties.

Recently, there have been active explorations into the role of TDP-43 in the viability and functions of vascular endothelial cells across various models (Xu et al., 2022; Hipke et al., 2023; Omar et al., 2023; Arribas et al., 2024). In this study, for the first time, we used the patient-derived EECM-BMEC-like cells to investigate this phenomenon morphologically and functionally. Consistent with previous findings, EECM-BMEC-like cells derived from a patient with *TARDBP* ^N345K/+^ displayed cytoplasmic mislocalization of TDP-43. Treatment with a chemical activator of Wnt/β-catenin signaling restored BMEC abnormalities in terms of disrupted tight junctions, barrier impedance and permeability, and VCAM-1 expression in ALS patient (*TARDBP* ^N345K/+^)-derived EECM-BMEC-like cells. We have previously reported that Wnt/β-catenin signaling activation promotes and reinforces the barrier properties of endothelial progenitor cells (EPCs) (Gastfriend et al., 2021b). Since EPCs develop a robust barrier function during differentiation into EECM-BMEC-like cells (Nishihara et al., 2020), Wnt/β-catenin signaling activation does not further enhance barrier function at this stage (passage 3–5), which is distinct from the plastic EPC stage (Gastfriend et al., 2021b). Indeed, administering Wnt activators to EECM-BMEC-like cells derived from gene-edited clones (*TARDBP* ^K263E/K263E^ and *TARDBP* ^G295S/G295S^) did not reinforce barrier function in the current experiment. These results suggest that Wnt/β-catenin signal activation may be effective only in damaged BMEC, making it a promising therapeutic approach for repairing BMEC, even in already matured brain endothelial cells.

The ALS patient harbored heterozygous SNP mutation at the coding exon six of *TARDBP,* resulting in a missense mutation (p.N345K) reported at the site known to be involved in the interaction of TDP-43 with other proteins (Rutherford et al., 2008). In this study, we also used *TARDBP* ^G295S/G295S^ and *TARDBP* ^K263E/K263E^ gene-edited hiPSCs, which mutations are known to be pathogenic that cause ALS and/or FTLD (Floris et al., 2015). We have previously reported that differentiated neurons using the same hiPSC lines with *TARDBP* ^K263E/K263E^ show a loss of function of TDP-43, indicating its usefulness in studying the impact of the mutation (Imaizumi et al., 2022). We asked if the specific *TARDBP* mutations contribute to BMEC dysfunction, however, *TARDBP* ^G295S/G295S^ and *TARDBP* ^K263E/K263E^ gene-edited hiPSCs-derived EECM-BMEC-like cells didn’t show impaired diffusion barrier functions nor enhanced immune cell adhesion compared to the isogenic control same as the ALS patient (*TARDBP* ^N345K/+^)-derived clone. The discrepancy between an ALS patient (*TARDBP* ^N345K/+^) and gene-edited clones (*TARDBP* ^G295S/G295S^ and *TARDBP* ^K263E/K263E^) might be the difference in the site of mutation (Figure 1A) or the type of allele mutation (heterozygous vs. homozygous). Indeed, the locus of mutation is associated with their aggregative capacity (Johnson et al., 2009), and the difference in vulnerability of TDP-43 between the locus of mutations might reflect the different phenotype between the mutations in our model. An alternative hypothesis is that the locus of mutations may be associated with cell-type-specific pathogenicity. This implies that the mislocalization of TDP-43 may occur in specific cell types depending on the locus of mutation. Indeed, *TARDBP* ^N345K/+^ was reported to show mislocalization of TDP-43 observed mainly in astrocytes than neurons, which is atypical compared to sporadic ALS patients, where TDP-43 aggregates are predominant in neurons (Takeda et al., 2019). Previously, Imaizumi and colleagues used the same iPSC clone carrying *TARDBP* ^K263E/K263E^ and reported that the TDP-43 functions were impaired in differentiated neurons, but preserved in neural progenitor cells and iPSCs themselves (Imaizumi et al., 2022). We cannot conclude whether *TARDBP* ^G295S/G295S^ and *TARDBP* ^K263E/K263E^ don’t show impaired TDP-43 functions in endothelial cells, but the alteration of BMEC functions could appear to depend on the site of mutation in *TARDBP*.

In the current study, we have also newly established the method of studying immune cell interaction with BMEC-like cells in the 96-well plate. The conventional assay could only compare a small number of wells, such as 8–16 wells (Nishihara et al., 2020; Nishihara et al., 2021), but the immune cell status could change from experiment to experiment, making it problematic to compare experiments performed on different days. In addition, the distribution of adherent cells is not always equal, and there was a possibility of bias at the imaging site when the analyzer chose where to photograph. To address these issues, we have modified the protocol to create a more effective assay that compares multiple clones under the same conditions in an unbiased manner using an automated analysis system. The major strength of our unique iPSC-derived BMEC-like cells is that they combine robust barrier function with the expression of cell adhesion molecules necessary for immune cell trafficking (Nishihara et al., 2020). Incorporating the iPSC-derived model into this newly established multi-well assay allows us to further accelerate the study of the interaction between the BMEC-like cells and immune cells using multiple clones.

Our study has limitations. First, we have only analyzed one patient-derived model and cannot conclude whether the specific ALS-associated mutation (*TARDBP* ^N345K/+^) contributes to BMEC dysfunction. Therefore, we cannot exclude the possibility that genetic background is an additional factor in barrier failure independent of *TARDBP* mutations or acts as a co-factor with *TARDBP* mutations necessary to affect BMEC functions. In the future, functional analysis using an isogenic model of *TARDBP* ^N345K/+^ or a genetically repaired model of *TARDBP* ^N345K/+^ carriers will be required to elucidate the association between the specific mutation and BMEC functions. In addition, there is still a limited number of autopsy cases reported focusing on the link between specific mutations and NVU abnormalities in ALS patients. Determining whether BMEC defects in ALS patients vary based on specific genetic mutations, not only *TARDBP* mutations but also *C9orf72*, *SOD1* and *FUS,* remains an important question for future research. Second, we have demonstrated impaired vascular barrier properties mainly using monocultured EECM-BMEC-like cells, and other NVU-forming cells such as pericytes and astrocytes have not yet been included in this model. These cells also express *TARDBP* and can be affected by its mutations. It has not yet been elucidated what effect coculturing these mutant NVU-forming cells has on the vascular barrier properties. Third, we have shown in this model an impaired diffusion barrier and the potential of BMECs for immune cell infiltration into the CNS. However, we did not provide evidence that BMEC dysfunction directly contributes to neurodegeneration. For future studies, advancing the NVU model, which recapitulates blood flow and incorporates pericyte, astrocytes, neurons and microglia is needed to show direct evidence of how peripheral changes cause neurodegeneration. Nevertheless, current study demonstrates that our ALS patient (*TARDBP* ^N345K/+^)-derived EECM-BMEC-like cells recapitulate BMEC morphological changes in ALS brain autopsied samples. Confirmation of BMEC dysfunction in an *in vitro* environment devoid of other factors such as inflammation or neurodegeneration suggests that BMEC abnormalities are not a secondary consequence of advanced pathology but rather directly implicated in ALS pathogenesis.

In conclusion, we established an ALS-derived BMEC-like cell that is useful in studying diffusion barrier functions and functional cell surface adhesion molecules. Familial ALS patient-derived BMEC-like cell showed disruption of tight junctions resulting in increased permeability of small molecule tracers and increased expression of cell surface ICAM-1 and VCAM-1, functionally attracting immune cells. Thus, our novel ALS patient-derived BMEC-like cell is useful for studying the involvement of vascular barrier dysfunction in the pathogenesis of ALS and promoting therapeutic drug discovery targeting BMEC. The novel automated techniques for analyzing immune cell adhesion in multiple samples facilitate further study of immune cell-BMEC interactions.

## Data Availability

The original contributions presented in the study are included in the article/Supplementary Material, further inquiries can be directed to the corresponding author.

## Supplementary material

SUPPLEMENTARY FIGURE S1 Representative images of triple staining TDP-43 (red), VE-cadherin (green), and DAPI (blue) in HC2-derived EECM-BMEC-like cells. TDP-43 proteins colocalized in nuclei were stained purple. Scale bar = 30 μm.

SUPPLEMENTARY FIGURE S2 Left: The impedance of monolayered EECM-BMEC-like cells was shown. Cells derived from TARDBP mutated gene editing hiPSC clones (left: TARDBP K263E/K263E right: TARDBP G295S/G265S) were seeded on E-plate 16. Some wells were treated with 4 μM CHIR99021 (green) or DMSO control (red). The black arrow indicated the timing when CHIR99021 or DMSO control was added to each well. Each condition had at least three replicates. Right: Area under the curve of the cell index in each condition.

SUPPLEMENTARY TABLE S4 Primer list.
