# Supplementary figures and images for: Establishment of a novel amyotrophic lateral sclerosis patient (TARDBP N345K/+)-derived brain microvascular endothelial cell model reveals defective Wnt/β-catenin signaling: investigating diffusion barrier dysfunction and immune cell interaction
Source: Front Cell Dev Biol. 2024 Aug 15;12:1357204. doi: 10.3389/fcell.2024.1357204 (PMC11357944; doi:10.3389/fcell.2024.1357204)

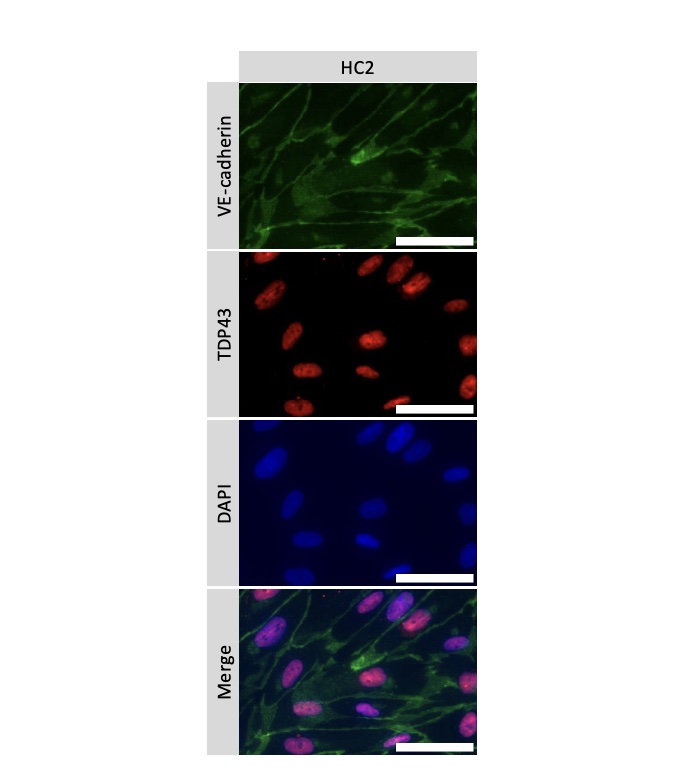

Supplement: Supplementary file 3 [file Image1.JPEG]

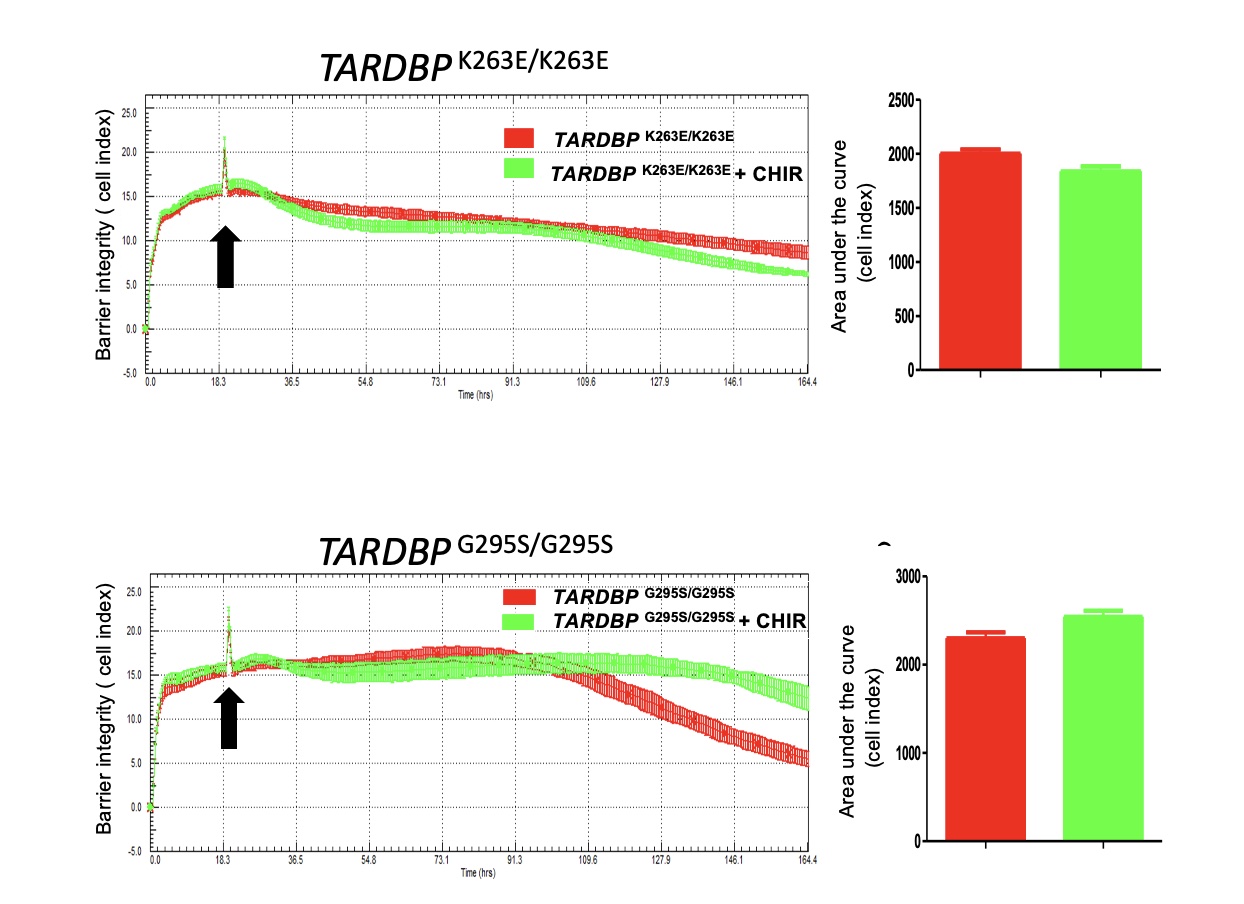

Supplement: Supplementary file 4 [file Image2.JPEG]
